# Depression among pet owners and non-pet owners: a comparative cross-sectional study in Dhaka, Bangladesh

**DOI:** 10.12688/f1000research.53276.1

**Published:** 2021-07-15

**Authors:** Samar Kishor Chakma, Taswib Tajwar Islam, Md. Shahjalal, Dipak Kumar Mitra

**Affiliations:** 1 Maternal and Child Health Division, International Centre For Diarrhoeal Disease Research, Dhaka, 1216, Bangladesh; 2 Department of Public Health, North South University, Dhaka, 1229, Bangladesh; 3 Public Health Nutrition, Primeasia University, Dhaka, 1213, Bangladesh; 4 Professor and Chairman, Department of Public Health, North South University, Dhaka, 1229, Bangladesh

**Keywords:** Pet and Depression, animal-assisted activities, pets and mental health, pets and psychological health

## Abstract

**Background: **Depression is a major contributor to overall global disease burden, often beginning in the teenage years and continuing into later life. Previous studies have reported high global rates of depression during these formative years, including in Bangladesh. At the same time, the therapeutic effect that pet ownership can have on depression is increasingly being recognized. However, studies examining these effects in Bangladesh are scarce. This study examined the association between household pet ownership and depression among people older than 13 years in Dhaka, Bangladesh.

**Methods:** A cross-sectional study was conducted using online and offline approaches. We used a snowball sampling technique to identify pet owners aged greater than 13 years residing in Dhaka, Bangladesh. Both web-based survey questionnaires using social media and hard copies were used to collect data from urban-dwelling pet owners. Logistic regression was used to identify the independent role of pet ownership in depression, adjusting for confounders including age, sex, marital status, known chronic disabilities, and other variables.

**Results:** A total of 140 pet owners and an equal number of non-pet owners participated in the study. Pet owners were found to be 41% less depressed than non-pet owners (AOR: 0.59; 95% CI: 0.31–1.14). In addition, males (AOR: 3.38; 95% CI: 1.50–7.62) who were either unmarried (AOR: 2.10; 95% CI: 1.05–4.16), smoked tobacco (AOR: 5.27; 95% CI: 1.50–18.53), or had a physical disability (AOR: 5.27; 95% CI: 1.50–18.53) were significantly more likely to be depressed.

**Conclusion: **Regression analysis revealed that in Dhaka, pet owners were associated with lower levels of depression compared to non-pet owners.

**Keywords**: Pets and depression, animal-assisted activities, pets and mental health, pets and psychological health.

## Introduction

Pet animals can play enormous roles in the lives of humans by providing company, support, and entertainment. There is evidence that the companionship of a household pet can improve human psychological health through the development of strong emotional bonds ( Wells, 2009). It has been observed that interaction with animals strengthens the human conscience in terms of behavior, attitude, responsibility, and social support ( Serpell, 1999). A survey of Americans aged 65 years and older revealed that in some groups, levels of depression were lower among pet owners than in non-pet owners, but the overall picture was complex ( Garrity, Stallones, Marx, & Johnson, 1989). Another study demonstrated that the presence of a resident dog for over two years reduced depression among the older residents of a nursing home ( Crowley-Robinson, Fenwick, & Blackshaw, 1996). Furthermore, a meta-analysis suggested that animal-assisted activities/therapy significantly reduces the symptoms of depression (standard deviation, SD = 0.61, 95% confidence interval, CI = 0.03–1.19) ( Souter & Miller, 2007). However, several research studies have also found that pet owners were in no better health than non-pet owners, or in some instances were even in worse health ( Herzog, 2011).

Depression is a challenging public health problem, and often first manifests in teenagers or those in their early 20s or 30s ( National Institute of Mental Health, 2015). The World Health Organization has long predicted that depression would become a leading contributor to disease burden in terms of disability-adjusted life years (DALY) calculated for all ages ( WHO, 2001). Currently, there are more than 300 million sufferers of depression worldwide; nearly half of these people live in South-East Asia and the Western Pacific Region ( WHO, 2017a). In Bangladesh, 4.1% of the population has a depressive disorder, responsible for 7.1% of the country's entire DALY ( WHO, 2017b).

Establishing a human–animal bond is just one of many well-known measures for treating depression. However, no study to date has examined this in Dhaka, Bangladesh. The principle aim of this study was to scrutinize the association between housing a pet animal and human depression in Dhaka. In particular, we compared depression in pet owners with that in non-pet owners, and analyzed other confounding associations with depression status.

## Methods

### Study design and participants

A cross-sectional study was conducted among the pet owners and non-pet owners. First, the sample size was calculated with the software Epi Info 7 (Centers for Disease Control). Based on previous findings, it was assumed that the minimal prevalence of depression among pet owners is 13% ( Sharpley *et al.*, 2020) and among non-pet owners is 36% ( Anjum, Hossain, Sikder, Uddin, & Rahim, 2019). Therefore, the alpha error was set to 5%, and the power of the study was set at 99%. According to the Fleiss with CC formula, 133 participants were required in each group, which increased to 140, and thus a total of 280 participants were to be recruited.

The eligibility criteria for both groups were that participants must be aged 13 years old or above and live in Dhaka, an urban area in Bangladesh. Participants who refused to give informed consent to participate were excluded from the study.

To maximize the number of responses we used both online and offline methods for collecting the data. Face-to-face interviews and a web-based survey ran simultaneously between December 2019 and March 2020. Each face-to-face interview lasted for 20 to 25 minutes.

The face-to-face interviews with pet owners were conducted in a veterinary clinic located within the study area. Pet owners were invited to ask their non-pet-owning friends who met the inclusion criteria to participate. In addition, non-pet-owning participants were randomly solicited in public locations (for example, tea stalls, educational institutions) to complete the offline questionnaire if they met the inclusion criteria.

The online questionnaire was advertised to members of pet-focused interest groups on Facebook. Interested individuals were directed to the questionnaire that was hosted on the web-based survey tool Google Forms.

After collecting data for one week—representing 20% of the non-pet owners and 13.57% of the pet owners—an analysis revealed no significant differences in the characteristics were observed between the data collected online and offline. Therefore, for reasons of cost-effectiveness and for convenience to the researchers, collection of data from both groups continued from January 2020 to March 2020 exclusively using the web-based survey.

## Ethical approval

Before data was collected, formal approval was granted by the Institutional Review committee of North South University, Dhaka, Bangladesh. Participants in the online survey responded anonymously and indicated their informed consent in the first section of the e-questionnaire; they were informed that completion of the questionnaire would denote the consent to participate in the study. Written informed consent was obtained before face-to-face interviews. Despite the methodology describing the minimum age as 13, all respondents (18 years and older) were adults in Bangladesh, therefore, parental/guardian’s consent was not sought.

## Questionnaire

The questionnaire was divided into two sections: sociodemographic questions and assessment of depression. Copies of the online and offline questionnaires are found in the *Extended data.*

*Section 1:* This section comprised a brief set of sociodemographic questions asking for the participant's current residence, age, gender, marital status (married, unmarried, divorced, or widowed), height, weight, monthly household income, employment status (job holder, business owner, homemaker, other,), educational status (primary or less, secondary, higher secondary, college degree and higher), and religion (Islam, Hinduism, Buddhism, Christianity).

This section also asked a few lifestyle-related questions on smoking and alcohol drinking habits (yes, no, or occasionally) and physical disability that leaves them unable to work (yes or no). Finally, this section asked whether or not participants owned a pet; those indicating at least one pet were further asked what type of pet they owned, the purpose of owing the pet, and for how long had they owned that pet.

*Section 2:* Depression was measured using the Patient Health Questionnaire 9 (PHQ-9) depression scale. PHQ-9 is scored from the Primary Care Evaluation of Mental Disorders Patient Health Questionnaire (PRIME-MD PHQ). It consists of nine questions aimed at detecting the symptoms of depression and a question to assess functional impairment. The authors of a validation study concluded that PHQ-9 is a reliable and accurate measure of depression severity and significance from a clinical and research perspective ( Kroenke, Spitzer, & Williams, 2001).

The responses to each question on the questionnaire are scored on a scale from 0 to 3 to give a total score ranging from 0 to 27. The responses to the questions indicate the occurrence of depressive symptoms over the previous two weeks with or without household pets. Responses were coded as 0 = not at all; 1 = several days; 2 = more than half the days; 3 = nearly every day. The total score represents the status of depression: <9 = absence of depression, ≥10 = presence of depression ( Manea, Gilbody, & McMillan, 2012).

## Data analysis

The dataset template was designed using IBM SPSS (Statistical Package for the Social Sciences) version 21, and analysis was performed using STATA 15 (Software for Statistics and Data Science). Cross tabulation was used to compare characteristics between the groups. A chi-square test was used with a significance level set at alpha <0.05 to determine the percentages and p-values for the categorical variables. An independent sample t-test was used to compare the means of all continuous variables between the groups.

A binary logistic regression was used to estimate the odds ratio with a 95% confidence interval to determine the odds that a participant would meet the criteria for depression status based on current pet ownership, controlling for age, gender, marital status, BMI status, monthly household income, occupation, education, religion, use of tobacco and alcohol, physical disability, and difficulty in working.

For analysis, some variables were categorized as dichotomous due to the small sample size. These variables were age group (>30 years, <30 years), marital status (married, unmarried), monthly household income (>60000 BDT, ≥60000 BDT), education (>12 years, others), religion (Muslim, others), tobacco use (yes, no), alcohol consumption (yes, no), difficulty in working, taking care of things or getting along with people with respect to PHQ-9 responses (yes, no).

## Results

A total of 290 participants enrolled in this study. Of these, 10 were omitted for giving incomplete answers, therefore the final number of respondents was 280, comprising 140 pet owners and 140 non-pet owners, thus maintaining the 1:1 ratio of group sizes. Table 1 shows the mean and standard deviation age and PHQ-9 scores for the pet owners and non-pet owners taking part in this study.

**Table 1.** Mean and standard deviation of participant age and PHQ-9 score.

Variables	Pet Owners (n = 140)	Non-pet owners (n = 140)	p-value
Mean age, (SD)	26.61 (6.18)	26.52 (5.41)	0.901
Mean PHQ-9 Score, (SD)	7.49 (5.88)	8.33 (6.01)	0.236

Table 2 lists the background characteristics, behavioral factors, disability status, and depression classification by pet ownership group. There were more female participants than male participants among pet owners (68.6% vs 31.4%), whereas there were more males than females for the non-pet owners (52.1% vs 47.8%). Marital status, age, religion, education level, tobacco and alcohol consumption, and monthly household income did not differ significantly between groups (p ≥ 0.05). Job holders constituted 34.3% of pet owners and 61.4% of non-pet owners in terms of occupation. “Other occupation” was reported by 45.7% of pet owners and 29.3% of the non-owning group. According to the data on religion, 93.6% of pet owners and 90.0% of the non-pet owners are Muslim, whereas in both groups, a small proportion (pet owners = 6.4%, non-pet owners = 10.0%) were of other religious denominations. A similar educational status was recorded for both groups, and more than 80% of the participants had >12 years of schooling.

**Table 2.** Sociodemographic characteristics of the participants.

Variable	Group				p-value
	Pet owner (n = 140)		Non-pet owner (n = 140)		
	n	%	n	%	
Gender					<0.001
Male	44	31.4	73	52.1	
Female	96	68.6	67	47.8	
Marital status					0.320
Married	39	27.9	50	35.7	
Unmarried	96	68.6	88	62.9	
Divorced	4	2.9	1	0.7	
Widowed	1	0.7	1	0.7	
Age group					0.647
≤30 years	115	82.1	112	80.0	
>30 years	25	17.9	28	20.0	
Monthly household income (Bangladeshi Taka)					0.290
<60000	96	68.6	104	74.3	
≥60000	44	31.4	36	25.7	
Occupation					<0.001
Job Holder	48	34.3	86	61.4	
Business owner	18	12.9	6	4.3	
Homemaker	10	7.1	7	5.0	
Other	64	45.7	41	29.3	
Religion					0.276
Islam	131	93.6	126	90.0	
Others	9	6.4	14	10.0	
Educational level					0.128
>12 years schooling	115	82.1	124	88.6	
<12 Years Schooling	25	17.9	16	11.4	
Tobacco consumption					0.221
Yes	32	22.9	41	29.3	
No	108	77.1	99	70.7	
Alcohol consumption					0.354
Yes	19	13.6	14	10.4	
No	121	86.4	126	90.0	
Physical disability					0.626
Yes	8	5.7	10	7.1	
No	132	94.3	130	92.9	
Depression					0.248
Depressed	40	28.6	49	35.0	
Non depressed	100	71.4	91	65.0	
Difficulty in working, taking care of things or getting along with people with respect to PHQ-9 responses					
Yes	82	58.6	62	44.3	0.017
No	58	41.4	78	55.7	
BMI status					0.019
Underweight	21	15.0	7	5.0	
Normal weight	76	54.3	73	42.1	
Overweight	32	22.9	43	30.7	
Obese	11	7.9	17	12.1	

The tobacco consumption rate among pet owners was 22.9%, and among non-pet owners, it was 29.3%. In total, 13.6% of the pet owners consume alcohol, whereas 10.0% of the non-pet owners drink alcohol. Only 5.7% of the pet owners reported having a physical disability, whereas for non-pet owners, the proportion was 7.1%.

There was a statistically significant difference in BMI status between the groups. The underweight rate was three times higher among pet owners (15.0%) than in non-pet owners (5.0%). The proportion of overweight pet owners was 22.9%, and 30.7% for non-pet owners. Regarding PHQ-9 responses, 41.4% of pet owners and 55.7% of non-pet owners reported difficulty working, taking care of things, or getting along with people. Moreover, 7.9% of the pet owners were obese compared to 12.1% of non-pet owners.

**Figure 1.**
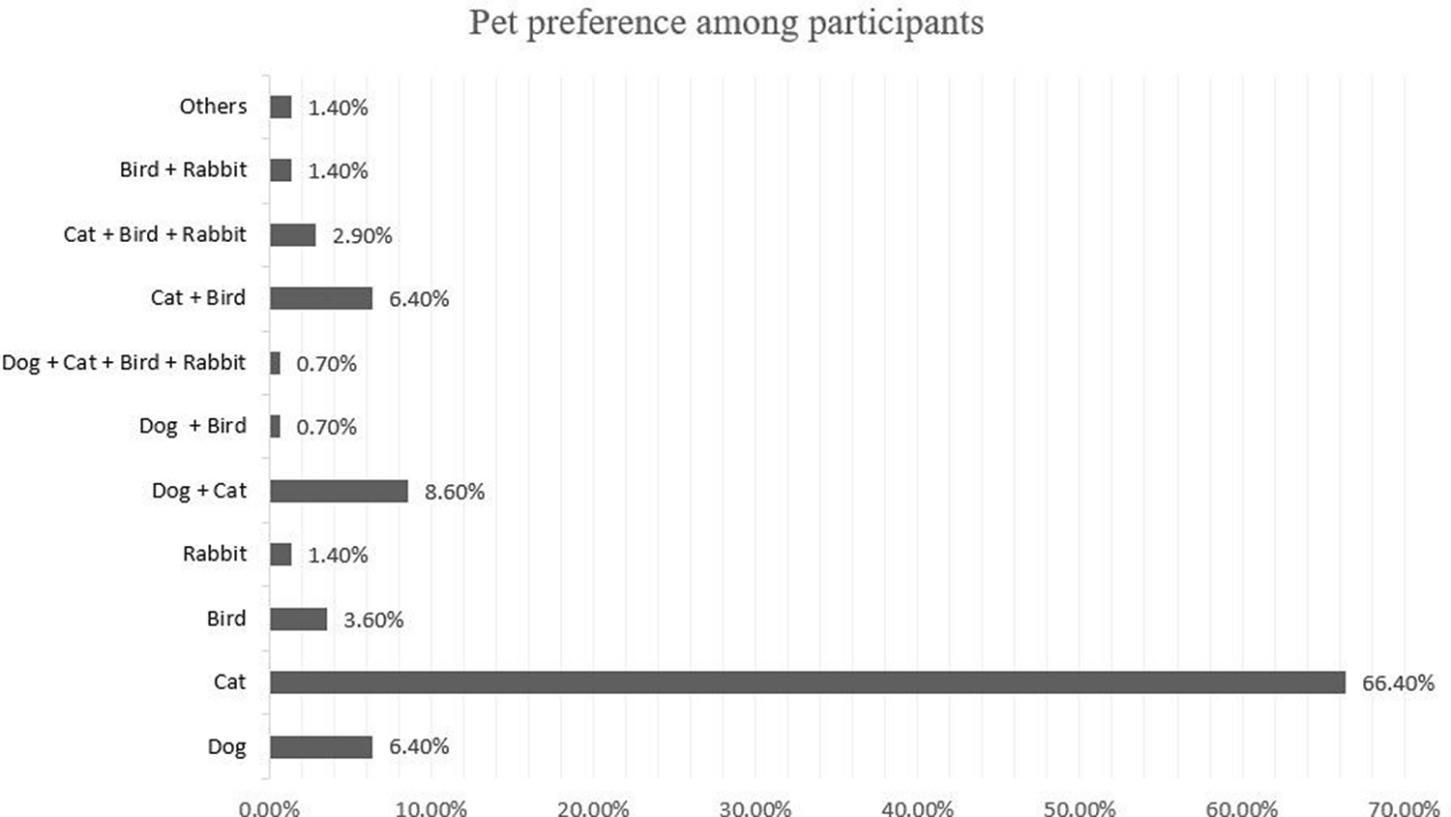
Pet preference among participants.

A detailed breakdown of pet ownership showed that 66.4% were cat owners, whereas 6.4%, 3.6%, and 1.4% owned dogs, birds, and rabbits, respectively. Other participants had more than one type of pet ( Figure 1).

**Figure 2.**
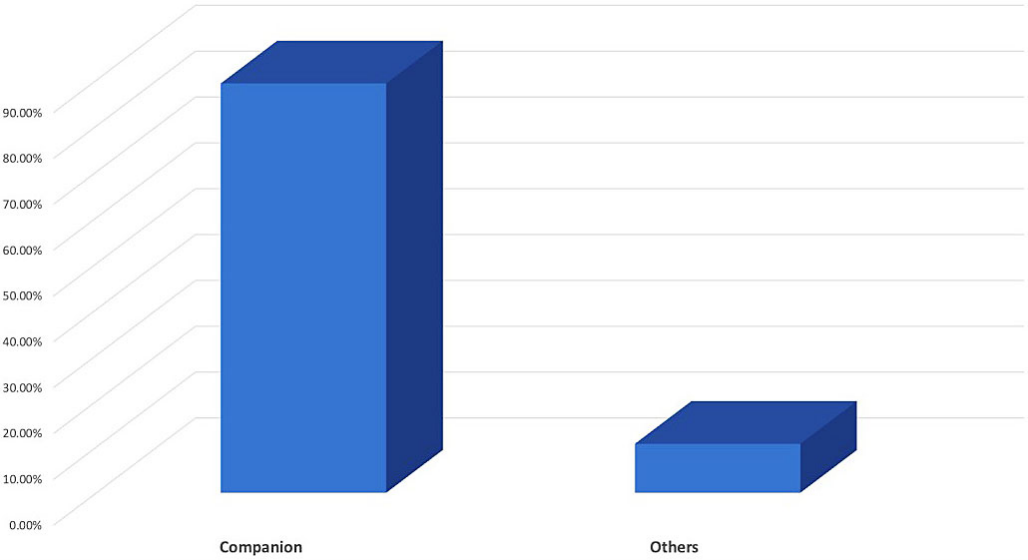
Purpose of having pets.

In terms of priorities for choosing a pet, 89.3% of owners keep pets primarily for companionship ( Figure 2). A total of 70.7% (n = 99) of the pet owners had owned their pet for <75 months, whereas the rest 29.3% (n = 41) had owned their pet for longer ( Figure 2).

Table 3 outlines the results of the bivariate logistic regression with a 95% confidence interval. Of the 13 predictor variables, six were statistically significant: gender (AOR: 3.38, CI: 1.50–7.62), marital status (AOR: 2.10, CI: 1.05–4.16), tobacco use (AOR: 2.72, CI: 1.04–7.09), physical disability (AOR: 5.27, CI: 1.50–18.53) and difficulty in working, taking care of things or getting along with people with respect to PHQ-9 responses (AOR: 5.94, CI: 3.16–11.16). Although not significant in this analysis, pet owners were 41% (AOR: 0.59, CI: 0.31–1.14) less depressed than the control group.

**Table 3.** Bivariate logistic regression predicting depression status.

Variable	Unadjusted OR (95% CI)	Adjusted OR (95% CI)
Pet owner		
No (reference)	1	1
Yes	0.74 (0.44–1.23)	0.59 (0.31–1.14)
Age group		
>30 years (reference)	1	1
<30 years	1.13 (0.60–2.13)	1.15 (0.54–2.43)
Gender		
Female (reference)	1	1
Male	2.04 (1.20–3.49)	3.38 (1.50–7.62)
Marital status		
Married (reference)	1	1
Unmarried	1.56 (0.89–2.75)	2.10 (1.05–4.16)
BMI status		
Obesity (reference)	1	1
Under weight	2.6 (0.83–8.07)	3.13 (0.73–13.6)
Normal weight	1.38 (0.54–3.47)	1.36 (0.43–4.32)
Overweight	1.24 (0.46–3.34)	1.39 (0.41–4.72)
Monthly income (Bangladeshi taka)		
<60000 (reference)	1	1
≥60000	1.43 (0.83–2.47)	1.14 (0.58–2.23)
Occupation		
Job holder (reference)	1	1
Business owner	1.87 (0.76–4.58)	3.08 (0.99–9.54)
Homemaker	1.83 (0.65–5.17)	1.99 (0.58–6.76)
Others	1.31 (0.75–2.28)	1.16 (0.58–2.28)
Education		
> 12 yearsschooling (reference)	1	1
<12 Years schooling	1.53 (0.71–3.27)	1.91 (0.77–4.74)
Religion		
Islam (reference)	1	1
Others	1.74 (0.62–4.86)	1.79 (0.55–5.77)
Tobacco		
No (reference)	1	1
Yes	1.07 (0.60–1.89)	2.72 (1.04–7.09)
Alcohol		
Yes (reference)	1	1
No	1.08 (0.49–2.38)	1.65 (0.49–5.49)
Physical disability		
No (reference)	1	1
Yes	3.70 (1.38–9.91)	5.27 (1.50–18.53)
Difficulty in working, taking care of things or getting along with people with respect to PHQ-9 responses		
No (reference)	1	1
Yes	4.96 (2.83–8.67)	5.94 (3.16–11.16)

## Discussion

This survey explored the association of pet ownership with depression. It was hypothesized that pet owners are less likely to be depressed than non-pet owners. We found that after adjusting for related confounders that pet owners were 41% less likely to be depressed than non-pet owners, although the result was not statistically significant. There is evidence supporting the association between pet ownership and lower level of depression. Garrity *et al.* (1989) found a positive impact of pet ownership on depression, emotional health, and physical health. Pet owners were found to be less depressed and had suffered fewer recent illnesses than the control group. Having a strong attachment with a pet was significantly (p = 0.0133) associated with less depression among older people (65+ years of age). McConnell *et al.* (2011) observed that pet ownership (specifically dogs) brings wellbeing to people who have social needs to fulfill and that these people were found to be less depressed.

However, in another study, pet ownership and attachment were not associated with depression and levels of loneliness in individuals living alone ( Antonacopoulos & Pychyl, 2010). Our study did not include a separate analysis to determine the singular associations based on the type of pet, as more than half of the participants owned cats (n = 93; 66.4%). Cline found no association between dog ownership and depression, but dog ownership was associated with greater wellbeing in married women and single adults ( Cline, 2010). Similarly, in another study, pet owners were found to have higher levels of satisfaction in life than non-pet owners, although they did not differ in happiness or positive or negative emotions compared to the other group. The length of time a primary pet is kept might be one reason for this opposing outcome as the average time was 5.5 years (SD = 3.88 years, range = 3 months–17 years) ( Bao & Schreer, 2016).

In this study, male participants have significantly higher (3.38 times) chances of being depressed than females. Similarly, Tower and Nokota (2006) found a lower level of depressive symptoms among unmarried women who lived with a pet than unmarried women who did not live with a pet. Consistent with our study, male participants have more depression than female participants, although no combined associations with depression were observed among sex, marital status, and pet ownership.

## Limitations

We acknowledge that this study had some limitations. The sample size was minimal. There is a possibility of recall bias among the participants while reporting information. In this cross-sectional study, the identified factors are regarded as associated factors, that is, either the causes or the results of depression. However, using a validated screening e-questionnaire was considered a cost-effective approach to exploring the general situation, and was therefore used in this study. The study population was from a township in an urban area of Dhaka, so the results may not be generally applicable to other urban or rural areas.

## Conclusion

The principal aim of this study was to determine the association between pet ownership and depression in Dhaka, Bangladesh. It was hypothesized that pet ownership is associated with fewer symptoms of depression. Logistic regression analysis indicated that pet owners were 41% less depressed than non-pet owners. In addition, some significant associations were found between depression and other variables, including gender, marital status, tobacco consumption, physical disability, and difficulty in working due to depressive symptoms. Considering these findings, more research is needed to investigate if pets cause depression and stress in both rural and urban contexts. Additionally, the current study can be further investigated through a qualitative approach.

## Data availability

### Underlying data

Mendeley Data: Depression among Pet owners and Non-pet owners: A comparative cross-sectional study in Dhaka, Bangladesh. http://doi.org/10.17632/bvrzf2s9j7.5 ( Chakma *et al.*, 2021).

This project contains the following underlying data:

- Data version 5 .sav

### Extended data

Mendeley Data: Depression among Pet owners and Non-pet owners: A comparative cross-sectional study in Dhaka, Bangladesh. http://doi.org/10.17632/bvrzf2s9j7.5 ( Chakma, *et al.*, 2021).

This project contains the following extended data:

- A copy of online questionnaire
- A copy of offline questionnaire

Data are available under the terms of the Creative Commons Attribution 4.0 International license (CC-BY 4.0).
