# Supplementary material for: Depression among pet owners and non-pet owners: a comparative cross-sectional study in Dhaka, Bangladesh
Source: F1000Res. 2022 Sep 7;10:574. Originally published 2021 Jul 15. [Version 2] doi: 10.12688/f1000research.53276.2 (PMC8383123; doi:10.12688/f1000research.53276.2)
Supplement: Peer review history

# Peer review: Reviewer response for version 2

**DOI:** 10.5256/f1000research.137902.r149773

**Published:** 2022-09-14

**Authors:** Caitlin R. Lisk

**Affiliations:** 1 University of Kansas Medical Center, Kansas City, KS, USA

I accept the revisions, thanks!

Is the work clearly and accurately presented and does it cite the current literature?

Partly

If applicable, is the statistical analysis and its interpretation appropriate?

Partly

Are all the source data underlying the results available to ensure full reproducibility?

Partly

Is the study design appropriate and is the work technically sound?

Yes

Are the conclusions drawn adequately supported by the results?

Partly

Are sufficient details of methods and analysis provided to allow replication by others?

Partly

Reviewer Expertise:

NA

I confirm that I have read this submission and believe that I have an appropriate level of expertise to confirm that it is of an acceptable scientific standard.

# Peer review: Reviewer response for version 1

**DOI:** 10.5256/f1000research.56642.r91335

**Published:** 2021-08-23

**Authors:** Jaymie Lynne Vandagriff

**Affiliations:** 1 Department of Human Development, Washington State University, Pullman, WA, USA

This study lends insight into the associations between pet ownership, depression status, and several other health-related variables among adults aged 18+ in an urban township of Dhaka, Bangladesh. The methods, results, and conclusions are sound and presented in a straightforward manner. I believe this study adds to the current literature by addressing a gap in the populations typically examined in the human-animal interaction literature (i.e., most studies have sampled participants in the US and UK and rarely include Muslim participants, to my knowledge) and adds to the literature on outcomes associated with cat ownership, as the participants were predominantly cat owners.

 I marked “Partly” regarding the presentation of methods/analysis because while all relevant statistics were reported for the main findings regarding Depression status, similar statistics were not consistently reported for the demographic variables. These are the only edits I would consider critical to revise for scientific soundness: the rest of this paper appears to be sound.

 Here are my comments per section:

- **Methods, page 3,** **paragraph 9**: regarding the line “…representing 20% of the non-pet owners and 13.57% of the pet owners”, it is unclear to what totals the 20% and 13.5% are referring. In addition, please report the statistical analysis conducted along with the specific statistics (e.g., t-test with *t*, sample size, df, and *p* values, or F-test with *F,* sample size, df, and *p *value) to demonstrate the data were equivalent.
- **Results, page 5, paragraph 1-5: **For this section which lists the between-group comparisons for demographic variables, please report the Chi-square or t-values for each comparison either in the text or in Table 2.

I have a few other suggested edits which are optional, but may strengthen the paper if the authors choose to incorporate them:

- **Introduction, page 3, paragraph 1:** regarding the line “…strengthens the human conscience in terms of behavior, attitude, responsibility, and social support”, consider changing “ conscience” to “consciousness" or "condition" as the listed examples do not appear related to one's sense of right and wrong.
- **Introduction, page 3, paragraph 3: **regarding the line “…is just one of many well-known measures for treating depression,” consider changing “ measures” to "avenues" or "methods" to describe the human-animal bond more clearly as a treatment approach rather than a metric.
- **Methods, page 3, paragraph 6: **Clarify whether the questions asked during the face-to-face interviews were identical to those presented in the data/extended data and Questionnaire section. It is also worth mentioning explicitly in this paragraph (or another) that a full version of the online and offline questionnaires are available and posted with the study data in this article, since this is a strength of the paper.

Great work overall.

Is the work clearly and accurately presented and does it cite the current literature?

Yes

If applicable, is the statistical analysis and its interpretation appropriate?

Yes

Are all the source data underlying the results available to ensure full reproducibility?

Yes

Is the study design appropriate and is the work technically sound?

Yes

Are the conclusions drawn adequately supported by the results?

Yes

Are sufficient details of methods and analysis provided to allow replication by others?

Partly

Reviewer Expertise:

Human-animal interaction; stress; mental health

I confirm that I have read this submission and believe that I have an appropriate level of expertise to confirm that it is of an acceptable scientific standard, however I have significant reservations, as outlined above.

# Response

**Published:** 2022-08-27

**Authors:** Samar Kishor Chakma

**Affiliations:** International Centre For Diarrhoeal Disease Research, Bangladesh

Thank you for your feedback and suggestions. We are grateful for your positive remarks about our article. We have considered your questions and concerns and have made the following changes. Reviewer Comments; followed by the response (in bold) by Authors:

**Methods, page 3, paragraph 9:** regarding the line “…representing 20% of the non-pet owners and 13.57% of the pet owners”, it is unclear to what totals the 20% and 13.5% are referring. In addition, please report the statistical analysis conducted along with the specific statistics (e.g., t-test with t, sample size, df, and p values, or F-test with F, sample size, df, and p value) to demonstrate the data were equivalent.

- **Thank you for bringing this issue to our attention. It was 20% of the total non-pet owners and 13.57% of the total pet owners.** **This statistical analysis can be found in the extended data section at http://doi.org/10.17632/bvrzf2s9j7.6 with all the information you requested showing that there were no significant differences between data collected online and offline. The manuscript has also been revised to clarify this point.**

**Results, page 5, paragraph 1-5:** For this section which lists the between-group comparisons for demographic variables, please report the Chi-square or t-values for each comparison either in the text or in Table 2.

- **Your feedback is greatly appreciated. In Table 2, page 6, p-values for each comparison based on the Chi-square test are presented.**

**Introduction, page 3, paragraph 1:** regarding the line “…strengthens the human conscience in terms of behavior, attitude, responsibility, and social support”, consider changing “conscience” to “consciousness" or "condition" as the listed examples do not appear related to one's sense of right and wrong.

- **Thank you for pointing that out. We have changed "conscience" to "consciousness".**

**Introduction, page 3, paragraph 3: **regarding the line “…is just one of many well-known measures for treating depression,” consider changing “measures” to "avenues" or "methods" to describe the human-animal bond more clearly as a treatment approach rather than a metric.

- **Thank you. We have changed the word "measures" to "methods".**

**Methods, page 3, paragraph 6: **Clarify whether the questions asked during the face-to-face interviews were identical to those presented in the data/extended data and Questionnaire section. It is also worth mentioning explicitly in this paragraph (or another) that a full version of the online and offline questionnaires are available and posted with the study data in this article, since this is a strength of the paper.

- **I appreciate you bringing this to our attention. It has been now clarified in the methods section that the online and offline (face-to-face interviews) interviews were identical. Moreover, we have indicated that a full version of both questionnaires is available in the extended data section.**

# Peer review: Reviewer response for version 1

**DOI:** 10.5256/f1000research.56642.r90883

**Published:** 2021-08-19

**Authors:** Caitlin R. Lisk

**Affiliations:** 1 University of Kansas Medical Center, Kansas City, KS, USA

- In the introduction, animal assisted therapy is referenced as a way to reduce symptoms of depression. Outcomes of therapeutic interventions are important, but because the focus of this article is on animal ownership, I would caution intermingling the two types of human-animal interactions.
- I would like to know the rate of animal ownership in Dhaka - is it more or less than the rest of the world? Who is most likely to own animals? What types are most owned?
- I appreciate that the questionnaire was provided and explained in detail. However, as a reviewer, I was unable to see the questionnaire or the data provided. 
- I would re-title Figure 1 to say "pet ownership" vs pet preferences. It could be that you interviewed all participants and asked what their preferences would be, including the non-pet owning group. 
- In reference to the discussion, are there national/worldwide statistics that you can reference in terms of depression?
- In the conclusion, you want to investigate whether pets "cause" depression and stress? Is there literature backing the claim that pets can cause depression? 
- Overall, I think this is a good study and I appreciate the authors submitting the article without significant results. This should happen more. However, the abstract and conclusion do not show that these are not statistically significant results, which I think is important for transparency. 

Is the work clearly and accurately presented and does it cite the current literature?

Partly

If applicable, is the statistical analysis and its interpretation appropriate?

Partly

Are all the source data underlying the results available to ensure full reproducibility?

Partly

Is the study design appropriate and is the work technically sound?

Yes

Are the conclusions drawn adequately supported by the results?

Partly

Are sufficient details of methods and analysis provided to allow replication by others?

Partly

Reviewer Expertise:

Human animal interaction

I confirm that I have read this submission and believe that I have an appropriate level of expertise to confirm that it is of an acceptable scientific standard, however I have significant reservations, as outlined above.

# Response

**Published:** 2022-08-27

**Authors:** Samar Kishor Chakma

**Affiliations:** International Centre For Diarrhoeal Disease Research, Bangladesh

We appreciate your feedback and suggestions. We are grateful for your positive remarks about our article. We have considered your questions and concerns and have made the following changes. Reviewer Comments; followed by the response (in bold) by Authors:

 In the introduction, animal assisted therapy is referenced as a way to reduce symptoms of depression. Outcomes of therapeutic interventions are important, but because the focus of this article is on animal ownership, I would caution intermingling the two types of human-animal interactions.

- **We have removed the reference to animal assisted therapy in the manuscript and clarified that the purpose of our study is to examine the benefits of pet ownership in managing depression, rather than explore the therapeutic effects of pets.**

I would like to know the rate of animal ownership in Dhaka - is it more or less than the rest of the world? Who is most likely to own animals? What types are most owned?

- **Thank you for bringing this issue to our attention. To our knowledge, there have been no surveys published online or in a record book regarding the prevalence of pet animal ownership in Dhaka. While we have attempted to find out information regarding your concern, no registry or record book has yet been compiled. **

** **


 I appreciate that the questionnaire was provided and explained in detail. However, as a reviewer, I was unable to see the questionnaire or the data provided. 

- **The questionnaire and the data can be accessed in the extended data section at the following link: http://doi.org/10.17632/bvrzf2s9j7.5**

** **


 I would re-title Figure 1 to say "pet ownership" vs pet preferences. It could be that you interviewed all participants and asked what their preferences would be, including the non-pet owning group. 

- **We have renamed Figure 1 to pet ownership vs pet preferences.**

** **


 In reference to the discussion, are there national/worldwide statistics that you can reference in terms of depression?

- **The statistics on depression have been added to the introduction paragraph number 2, thus we have not mentioned them in further detail in the discussion. Thank you for your suggestion. For future reference, we will keep this in mind.**

** **


 In the conclusion, you want to investigate whether pets "cause" depression and stress? Is there literature backing the claim that pets can cause depression? 

- **The conclusion has been edited to reflect that the purpose of our study is not to investigate whether pets "cause" depression and stress, but rather to examine whether pets can help manage depression. Thank you for bringing this to our attention. **

# Peer review: Reviewer response for version 1

**DOI:** 10.5256/f1000research.56642.r90915

**Published:** 2021-08-09

**Authors:** Shelly L. Volsche

**Affiliations:** 1 Department of Anthropology, Boise State University, Boise, ID, USA

The manuscript "Depression among pet owners and non-pet owners: a comparative cross-sectional study in Dhaka, Bangladesh" is a well written, concise research report on the findings of a survey conducted online and offline. It is well designed, with a clear control group. The results are clearly reported and the methods (including ethical review) are easy to follow and envision for replicability.

 If there is room, one to two more paragraphs could be added to the Discussion to further analyze the findings that pet owners were more likely to be underweight. This is particularly interesting since these individuals are primarily cat owners; hence, not likely taking their animals for daily walks. It may be worthwhile to consider what this means in the spectrum of broader health implications. Are these individuals (mostly Muslim women from the demographic data) single or economically vulnerable? Could this suggest why pet ownership is so beneficial for them?

 Either way, this is a well written research report that contributes important data to the "pet effect" discussion.

Is the work clearly and accurately presented and does it cite the current literature?

Partly

If applicable, is the statistical analysis and its interpretation appropriate?

Yes

Are all the source data underlying the results available to ensure full reproducibility?

No source data required

Is the study design appropriate and is the work technically sound?

Yes

Are the conclusions drawn adequately supported by the results?

Yes

Are sufficient details of methods and analysis provided to allow replication by others?

Yes

Reviewer Expertise:

human-animal interactions; kinship; evolutionary cognition/psychology; animal behavior.

I confirm that I have read this submission and believe that I have an appropriate level of expertise to confirm that it is of an acceptable scientific standard.
